# Mixed-methods study of medical students’ attitudes toward peer physical examinations in Japan

**DOI:** 10.1186/s12909-024-05635-4

**Published:** 2024-06-20

**Authors:** Emily Suzuki, Nobutoshi Nawa, Eriko Okada, Yu Akaishi, Ayako Kashimada, Mitsuyuki Numasawa, Kumiko Yamaguchi, Kazuki Takada, Masanaga Yamawaki

**Affiliations:** 1 Department of Medical Education Research and Development, Tokyo Medical and Dental University, 1-5-45 Yushima, Bunkyo-ku, Tokyo, 113-8519 Japan; 2 Department of Global Health Promotion, Tokyo Medical and Dental University, Tokyo, Japan; 3 Institute of Education, Tokyo Medical and Dental University, Tokyo, Japan; 4 Institute of Global Affairs, Tokyo Medical and Dental University, Tokyo, Japan

**Keywords:** Peer physical examination, Human rights protection, medical students in Japan, Incidental findings, PPE policy, autonomy, privacy

## Abstract

**Background:**

Most Japanese medical schools likely continue to rely on peer physical examination (PPE) as a tool to for teaching physical examination skills to students. However, the attitudes of medical students in Japan toward PPEs have not be identified. Therefore, we evaluated students’ attitudes toward PPE in a Japanese medical school as a preparation for developing a PPE policy tailored to the context of Japanese culture.

**Methods:**

We conducted a mixed-methods study with an explanatory sequential approach, in which qualitative data were used to interpret the quantitative findings. Surveys and interviews were conducted with medical students and junior residents at a Japanese university. A total of 63 medical students and 50 junior residents responded to the questionnaire. We interviewed 16 participants to reach theoretical saturation and investigated the attitudes of medical students toward PPE and the themes emerging from the interview data, providing detailed descriptions of the quantitative findings.

**Results:**

Female participants were significantly more likely than male participants to report varying degrees of resistance to being a model patient during PPE (male: 59.7%, female: 87%, *p* *<* 0.001). Most of the participants who took on the role of patients that involved undressing were males. The participants expected improvements in issues related to the guarantee of freedom to refuse to be a model patient and measures to protect confidentiality. Approximately 22% of the participants reported that they witnessed incidental findings (including variations within the normal range) in front of other students during PPE.

**Conclusions:**

The findings imply that medical students expect high levels of autonomy and confidentiality when volunteering as model patients during PPE. Thus, developing a PPE policy suitable for Japanese culture may be effective in establishing a student-centered PPE environment.

**Supplementary Information:**

The online version contains supplementary material available at 10.1186/s12909-024-05635-4.

## Background

Peer physical examination (PPE) is a training method in which students learn basic examination skills by practicing on fellow students or by being examined by tutors in front of other students. PPE became popular in the 1990s as an alternative educational method for the traditional bedside clinical training owing to changes in health care services such as shorter hospitalization periods, declining number of inpatients, greater patient understanding regarding the need for medical education, and increasing workload of hospital staff [1]. PPE has enabled medical students worldwide to improve their examination techniques despite limited access to real patients [2]. PPE also allows students to conduct repetitive trial-and-error practice until they gain confidence in their examination skills [3]. Moreover, it helps students learn basic anatomy and signs with standardized patients [4]. PPE requires students to take turns as model patients, thus offering students with an opportunity to receive feedback and learn about the patients’ feelings. Additionally, medical students worldwide have shown acceptance for PPE. For example, in the United States, Chang et al. reported a high level of acceptance rate (98%) for PPE at the University of Minnesota [5]. Rees et al. also stated that more than 83% of medical students accept PPE of non-intimate body parts in 6 medical schools around the world that are geographically and culturally different, including schools in Hong Kong and Japan [6]. Gupta et al. also reported high acceptance for PPE of throat among medical students in India in 2018 before and after PPE sessions (71.7% and 95.7%, respectively) [7]. However, for certain students, being a *patient* in front of their peers is unacceptable due to their cultural, religious, and personal backgrounds [4]. Sato et al. compared anatomy teaching methods between Japan and the United Kingdom and highlighted the possiblity that attitudes toward nakedness and the living body are deterrent to the widespread use of PPE in Japan [8]. Tagawa et al. administered a questionnaire surveyto students in a single medical school in Japan regarding their perception of PPE, finding that a few male students who strongly refuse to be examined. The authors concluded that such students should not be forced to become examinees only because they are male [9]. Additionally, scholars observed a same-gender preference regarding the examination of certain body parts such as the chest and inguinal region [5]. Despite the need for special consideration for PPE, volunteering as a simulated patient may become a coercive act if the active participation of students is assessed as a component of their course grades [10]. Furthermore, addressing medical problems discovered during PPE requires the protection of their privacy and the provision of appropriate healthcare services [11]. 

Numerous studies have discussed the ethical issues related to PPE in terms of students’ autonomy and privacy [5, 10–13]. To address the increased demand for human rights protection among medical students, the American Medical Association provides a code of conduct for PPE [14]. While Das et al. and Rees et al. have demonstrated the impact of the students’ ethnicity regarding their acceptance of PPE [6, 15], few studies have discussed the attitudes of students in Asian countries toward PPE.

Within the model core curriculum for medical education in Japan, which shapes approximately two-thirds of the curriculum of the Japanese medical schools, physical examination is listed as a basic skill in medical practice to be learned prior to clinical clerkship [16]. Among the educational resources for simulation-based learning, such as virtual reality simulators and plastic models, PPEs are free from financial restrictions and system operations [17–19]. Thus, there is a possibility that the majority of Japanese medical schools will continue to generally rely on PPEs as a tool to teach physical examination skills to students. Therefore, the attitudes of medical students in Japan toward PPEs should be assessed from the viewpoint of protecting the autonomy and privacy of students in improving their learning environment. Thus, this study aims to evaluate the attitudes of students in a Japanese medical school toward PPEs as a preparation for developing a PPE policy that is tailored to Japanese culture with reference to PPE policies overseas.

## Method

### Sample

The study conducted an online survey using an anonymous questionnaire via Google Forms. Specifically, a link to the online questionnaire was distributed to the whole 217 undergraduate medical students in their fifth and sixth years at TMDU (111 fifth grade students and 106 sixth grade students) between June 21 and July 31, 2022. The same questionnaire was sent to 190 junior residents of Tokyo Medical and Dental University (TMDU) Hospital in November 2022. They were requested to submit their responses online between December 4 and 13, 2022. The respondents had experienced PPEs at their medical schools. Supplementary Fig. 1 presents the eligibility criteria for quantitative analysis, and Supplementary Data A provides additional details on the educational setting. The researchers in this study included those who are responsible for medical education at the Tokyo Medical and Dental University and hold leadership positions in Medical Education in Japan. Based on their experience, we assumed a priori that 60% and 85% of male and female students were reluctant, in varying degrees, to be model patients for PPE. Using a two-tailed test with a significance level of 0.05 and a sample size of 98, the statistical power reached 80% with a margin of 10%; thus, a sample size of 109 or more was estimated to be sufficient.

Further, we sent an invitation for interviews via Google Form to all medical and junior residents on December 8, 2022, of which 14 and 2, respectively, showed interest in participating. Thus, we conducted individual interviews with the 16 participants to learn about their personal experiences with PPEs. Supplementary Fig. 2 (Supplementary Data B) describes the eligibility criteria for qualitative analysis. Informed consent was obtained from the participants prior to each interview. Supplementary Table 1 (Supplementary Data B) presents the characteristics of the participants who participated in the interviews. This study conforms to the standards for reporting qualitative research and observational studies [20, 21]. The Research Ethics Committee of TMDU approved the study (approval number M2021-141).

### Questionnaire survey

The study obtained information about the respondents’ demographic characteristics such as their year of schooling, year of junior residency, gender, and their perception toward PPEs. The students and junior residents specified the situational setting in which they experienced PPEs and their level of agreement with a statement regarding their views about PPEs. To ensure criterion validity, the item on willingness to be a model patient adapted a question used in a previous study performed in a Japanese university [9]. Furthermore, the researchers in this study included those who are responsible for medical education at Tokyo Medical and Dental University and hold leadership positions in Medical Education in Japan. We conducted a brief focus group discussion among the researchers in this study to determine the content of our questionnaire survey to ensure content and construct validity. Supplementary Data C provides details on the survey questions.

### Individual interviews

We interviewed 16 participants (7 male and 7 female medical students and 1 male and 1 female junior resident) until theoretical saturation was reached. Theoretical saturation was defined as the point at which further interviews no longer lead to additional data [22]. We considered that the interviews reached theoretical saturation when the responses from the last 4 interviewees were identical to those of the previous ones and failed to provide any additional insight. Moreover, interviewees and did not give us any additional insight. Moreover, 15 participants are generally considered sufficient for collecting data through interviews in qualitative research, thus, our judgement of data saturation can be further justified [23]. 

The interviews lasted for approximately 1 h and were conducted in Japanese face-to-face in a conference room at TMDU or via Zoom® using an interview guide (Supplementary data D). The interview guide included questions on their experience with PPEs, privacy protection measures, desirable procedures in the discovery of suspicious findings, and the value of the experience of being a model patient. The interviews were audio-recorded and transcribed verbatim. The transcript was analyzed with regard to the research themes.

### Data analysis

After the questionnaire survey was filled, the medical students and junior residents were invited for individual interviews. The study qualitatively analyzed their responses to the open-ended questions in the interview. Moreover, we used the explanatory sequential approach in which qualitative data were used to provide detailed descriptions of the quantitative findings [24]. The representative quotes derived from the qualitative phase were presented with the results of the quantitative data to integrate both results [24]. For data triangulation, we requested experts in the preclinical and clinical education of the medical students to verify the accuracy of our interpretations and ensure credibility [25]. 

Reflexivity was also considered during coding; [24] we tried not to make excessive interpretations of what was said. To ensure that key themes were not excluded and check the credibility of our interpretations, we engaged in member checking with three physician entrepreneurs with whom we conducted in-depth interviews and seven medical students with whom we conducted individual interviews by showing them the final results of our analysis [25]. All of them indicated that the interpretation resonated with their experiences.

## Results

### Demographic characteristics of the participants

Of 217 and 190 medical students and junior residents, respectively, 63 and 53 responded to the questionnaire. We excluded one junior resident who had graduated from a foreign university and two junior residents who did not supply the name of the university from which they graduated, resulting in a final sample size of 113 (Supplementary Fig. 1). The sample comprised 67 (59.3%) males and 46 (40.7%) females. Table 1 presents the characteristics of the respondents. All of them had experienced PPEs prior to the study.

**Table 1:** Characteristics of the participants (survey)

	Male	Female
Fifth-year students	19	12
Sixth-year students	19	13
First-year junior residents	18	14
Second-year junior residents	11	7
Total (*n* = 113)	67 (59.3%)	46 (40.7%)

### Demographic characteristics of the interviewees

Supplementary Fig. 2 (Supplementary Data B) shows that 2 of the 16 participants were junior residents, one of whom graduated from a university other than TMDU.

### Unwillingness to be examined in PPEs

Table 2 demonstrates that 40 of 67 male participants (59.7%) and 40 of 46 female participants (87%) expressed varying degrees of reluctance to be examined as patients in PPEs, with more females expressing unwillingness (*p* < 0.001).

**Table 2:** Unwillingness of the participants (current and previous medical students [i.e., junior residents]) to be examined as patients in PPEs that involve undressing and their actual previous experience of the patient role against their will

		Male participants (*n* = 67)	Female participants (*n* = 46)	df	*p*-value
Unwillingness	Not at all	27 (40.3%)	2 (4.3%)	(5)	**< 0.001**
	Rarely	18 (26.9%)	1 (2.2%)	(5)	
	Sometimes	16 (23.9%)	11 (23.9%)	(5)	
	Often	3 (4.5%)	10 (21.7%)	(5)	
	Always	3 (4.5%)	18 (39.1%)	(5)	
	Missing	0 (0%)	4 (8.7%)	(5)	
Actual previous experience against their will	No	48 (71.6%)	43 (93.5%)	(2)	**< 0.001**
	Yes	19 (28.4%)	1 (2.2%)	(2)	
	Missing	0 (0%)	2 (4.3%)	(2)	

Bold values indicate *p* < 0.05

Furthermore, in contrast to 1 female (2.2%), 19 (28.4%) males had been physically examined against their will (*p* < 0.001). The findings from the interviews supported this notion:

*“*Although I was not totally forced to be a model patient during the PPE class against my will, I was reluctant to play the role. The reason I accepted to be the model patient was because I had no choice but to do so due to the limited number of male students in the same group. I thought there was a perception that male students usually do not mind being a model patient in front of peers. Therefore, it was difficult for me to speak out about my reluctance to be a model patient during PPE.*”* [Male medical student (MMS) 1]

Supplementary Data E demonstrates that the unwillingness of the participants differed according to the body region under study. Supplementary Data F provides additional descriptions.

### Practice environment of PPEs

Table 3 showed that 67 male participants (100%) experienced the role of a patient, which involved undressing during PPEs, while 40 female participants (87.0%) never experienced this role (*p* < 0.001). A few female interview participants reported that they experienced the role of a patient in thyroid palpation, fundus examination, neurological examination, and hand joint examination, which did not require undressing.

**Table 3:** Responses to the peer physical examination questionnaire by gender

		Male participants (*n* = 67)	Female participants (*n* = 46)	Total	df	*p*-value
	1. Have you ever experienced the role of a patient that involves undressing in PPEs?					
Yes		67 (100%)	6 (13.0%)	73 (64.6%)	(1)	**< 0.001**
No		0 (0%)	40 (87.0%)	40 (35.4%)	(1)	
	2. Questions for men: What do you think about women playing the role of a patient in a PPE class?					
I think it is a good idea.		30 (44.8%)	NA	30 (44.8%)	NA	NA
I don’t think it is a good idea.		12 (17.9%)	NA	12 (17.9%)	NA	NA
I don’t know/No answer		25 (37.3%)	NA	25 (37.3%)	NA	NA
	3. Questions for women: What do you think about men playing the role of a patient in a PPE class?					
I think it is a good idea.		NA	23 (50%)	23 (50%)	NA	NA
I don’t think it is a good idea.		NA	5 (10.9%)	5 (10.9%)	NA	NA
I don’t know		NA	18 (39.1%)	18 (39.1%)	NA	NA
4. When you played the role of a patient, was your privacy protected?						
Yes		46 (68.7%)	13(28.3%)	59 (52.2%)	(3)	**< 0.001**
No		19 (28.4%)	7 (15.2%)	26 (23.0%)	(3)	
I don’t know.		2 (3.0%)	18 (39.1%)	20 (17.7%)	(3)	
No answer		0 (0%)	8 (17.4%)	8 (7.1%)	(3)	
	5. Do you feel resistance in performing PPE on a student whose gender is different from yours?					
Yes		20 (29.9%)	20 (43.5%)	40 (35.4%)	(1)	0.137
No		47 (70.1%)	26 (56.5%)	73 (64.6%)	(1)	
	6. There is a view that practicing auscultation and echocardiography skills is not necessary for students if they practice those using simulators. Do you agree or disagree with this view?					
Agree		15 (22.4%)	18 (39.1%)	33 (29.2%)	(2)	0.129
Disagree		50 (74.6%)	26 (56.5%)	76 (67.3%)	(2)	
Other		2(3.0%)	2 (4.3%)	4 (3.5%)	(2)	
	7. Prior to the PPE class, were you given guidelines regarding playing the patient role or did you sign a document confirming that you had the right to refuse to play the patient role?					
Yes		3 (4.5%)	2 (4.3%)	5 (4.4%)	(2)	0.690
No		43 (64.2%)	26 (56.5%)	69 (61.1%)	(2)	
I don’t remember.		21 (31.3%)	18 (39.1%)	39 (34.5%)	(2)	

Bold values indicate *p* < 0.05

During the interviews, one of the cited reasons for the fact that males comprised the majorityin the role of patient in PPE was that the teaching staff, especially male instructors, explicitly asked male medical students to be model patients, as confirmed by the following excerpt:

*“*During the PPE class, the teachers clearly stated that male students were encouraged to volunteer for the model patient. For example, when I practiced the procedure of blood test with a group of four (three female students and a male student), a male instructor directly asked the male student to be a model patient of the blood test, although there was no obstacle for female students to play the role of patient in blood test. I always envied male students because they could learn something from the experience of model patients*”* [ FMS6].

The female participant also mentioned the education gap between themselves and males in the following statement.

“I think there are lessons that medical students can learn only through the experience of being a model patient role. I was jealous of male students because there were limited chances for female students to learn the feelings of patients through PPEs.” [FMS6].

In contrast, 30 male participants (44.8%) considered that playing the role of a patient is inappropriate for females, and 23 female participants (50%) expressed that males playing the role of a patient is a good idea.

Among the participants who played the role of a patient, male participants were more satisfied with privacy protection than the female participants (68.7% vs. 28.3%, *p* < 0.001). The privacy concerns raised during the interviews ranged from compulsory model patient roles for male students to the inappropriate remarks of male students. This was shown in the following statements:

*“*I worried about the potential risk of forcing male students to be model patients.” [FMS7].

“I remember that male teachers sometimes mentioned a model patient’s physical characteristics. For example, a teacher praised a male student who volunteered for the abdominal examination for his toned abs. It might cause model patients who are out of shape to be uncomfortable.” [FMS2].

From the viewpoint of the examiner, 29.9% and 43.5% of the male and female participants, respectively, expressed unwillingness to perform examinations on peers of the opposite gender (*p* = 0.137). Although a few male participants showed support when female students volunteered as a model patient in the interview, others were cautious due to high psychological resistance among male students and the risk of false accusations.

“I appreciate female students for their active participation in PPE as a model patient. When I needed to examine a real female patient at the clinical clerkship, I had no idea where to put my stethoscope on her chest. Due to the difference in physical characteristics by gender, it is important to practice on female students in medical school.” [MMS3].

“I have a higher psychological resistance toward performing a physical examination on female classmates than performing the same examination on a patient after meeting them for the first time.” [MMS2].

*“*I don’t want to perform physical examination on female students to avoid false accusations of inappropriate touching.” [MMS5].

However, 67.3% of the participants (male: 74.6%; female: 56.6%) felt that practicing auscultation and echocardiography skills on a peer played an irreplaceable role in developing their skills. In the interview, the participants mentioned the benefits of PPE and the limitations of simulators as follows:

“Although simulator-based learning is helpful, we can only acquire some skills by practicing on living humans. For example, we cannot practice neurological examinations on simulators.” [FMS5].

“We cannot get feedback on our performance from simulators.” [MMS4].

Sixty-nine participants (61.1%) reported that no guidelines exist for PPE and no consent is obtained for the procedure, while five participants (4.4%) mentioned that they were shown a PPE guideline or asked to append their signature to an informed consent form.

### Request of improvements in PPEs

Table 4 indicated that 51 participants (54.8%) expected an assurance of the right to refuse to play the role of the patient regardless of gender. More than 30% of the participants requested rule-based measures in the case of a breach in confidentiality, combined with the introduction of confidentiality protection. Regarding the introduction of a procedure to obtain consent, an oral form gained more support than a written form (41.9% vs. 23.7%). Further, they pointed out that the consultation services established to address the anxiety of students in relation to PPEs need improvement.

**Table 4:** Aspects of PPEs that require improvement

Aspects of PPEs that require improvement (*n* = 93)	
Guarantee of the freedom to refuse to play the patient role regardless of gender	51 (54.8%)
Confidentiality	29 (31.2%)
Rule-based measures against a breach in confidentiality	44 (47.3%)
Introduction of a procedure (oral) for obtaining consent to play the patient role in a partial- or full-body examination	39 (41.9%)
Introduction of a procedure (written) for obtaining consent to play the patient role in a partial- or full-body examination	22 (23.7%)
Establishment of a consultation service	22 (23.7%)
Notification of abnormal findings during the PPE	1 (1.1%)

### Management of possible abnormalities found in students

Table 5 demonstrated that 25 participants (22.1%) experienced an incidental identification of physical abnormalities (e.g., variations within the normal range) in front of other students during PPEs. One participant noted that a model patient (i.e., a medical student) let a tutor and other students know about the possibility of abnormal findings in the particular body region prior to the examination. In addition, a tutor publicly pointed out the abnormality, which was known to the student. In other words, the student may have handled the risk of privacy violation by themselves to their tutor and other students.

**Table 5:** Responses to abnormal findings during PPE

What did you or your supervisor do when you accidentally found abnormal findings on the body of a student playing the role of a patient? (*n* = 113)	
Pointed it out publicly on the spot	25 (22.1%)
Informed the student of it confidentially	4 (3.5%)
There was no such situation, or I did not notice it	72 (63.7%)
I don’t remember or I don’t know./No answer	12 (10.6%)

A few male interview participants reported that they were confused about instructors’ comments on their physical characteristics during PPE.

*“*When an instructor said that my prostate was comparatively small, my classmates laughed. I did not care too much about the event, but there might be some students who would really care if the same situation happens to them.” [MMS1].

## Discussion

This study analyzed the perceptions of medical students and junior residents regarding PPEs from the following: (a) unwillingness to be examined, (b) practice environment, (c) improvements to be made, and (d) management of abnormal findings. We found that (a) more than half of the male and female participants expressed varying degrees of unwillingness to be model patients during PPEs with a significantly higher rate of unwillingness among the female participants. Additionally, 28.4% of the male participants were forced to experience the role of model patients against their will. This finding was almost identical to that of a previous study that reported that 24.5% of 6th year male medical students in Japan found being examined in a small group with female students during PPE embarrassing [9]. (b) Moreover, the study observed that male students comprised the majority of those who took on the role of patients that involved undressing. Nevertheless, only 50% of the female participants answered that being model patients is appropriate for males, while 44.8% of the males responded that participating in PPEs as patients is a good idea for females.(c) The participants expected improvements in providing guarantees of the freedom to refuse to be a model patient and confidentiality protection measures. (d) Only 22.1% of the participants reported that they witnessed incidental findings in front of other students during PPE.

The low levels of willingness of the female participants to take on the role of a patient is consistent with those of previous studies in the United States and the United Kingdom [26, 27]. However, in contrast to previous research that reports a high acceptability rate of PPE of non-intimate body parts in other countries, including India [5–7], the current study revealed students’ unwillingness to be a model patient during PPE, with participants expressing varying degree of willingness and unwillingness to be examined. Furthermore, more than 25% of the male participants experienced the role of a patient against their will, which is a notable issue. Expectedly, the male participants nearly exclusively played the role of a patient in PPE that involved undressing. Despite this situation, only half the female participants responded that playing the role of the patient is a good idea for males. The two issues that emerged for the fact that a majority of those who took on the role of model patients were males are related to the possibility of external pressure on them to play the role and the loss of learning opportunities for female students in terms of gaining awareness of the feeling of patients by experiencing this role. One of the possible reasons for this may be that a few of the female students feel guilty and a sense of injustice if they let the existing situation in which male students are forced to be model patients against their will purely due to their gender continue. This feeling may be shared among the majority of the medical students, because the majority of the participants expected improvements in the guarantee of the freedom to refuse to play the role of the patient regardless of gender as the leading task. Likewise, the fact that 44.8% of the male participants endorse females to play the role may imply a feeling of gender-based unfairness among males. Additionally, the limited opportunities for female students to be model patients may exert a negative impact on the educational value for both male and female students. Given that female students do not always feel a sense of security in privacy protection during PPEs, increasing the security level is an urgent matter to address. To enable students to acquire the skills necessary for examining female patients by taking into account their unique physical characteristics, requesting the cooperation of living models is a useful option [28]. Furthermore, if students witness senior students being examined by their peers at induction sessions, they may be encouraged to participate in PPE, thus avoiding the problem of the shortage of model patients. Previous studies have reported that students prefer to be examined by peers of the same gender [29]; thus, same-sex PPE can be considered as a way to compensate for the lack of learning opportunities. However, in light of gender diversity, special consideration is necessary when grouping students by gender, especially for students who do not wish to reveal their gender identity. The incidence rates of physical abnormalities identified in front of other students during PPE was 22.1%, which was higher than that of a medical school in the Netherlands (16. 8%) [11]. The reason might be that physical abnormalities that may require medical intervention and physical characteristics that were unique but within a normal range were also included in the incidental findings based on the responses in the interview. Given that commenting on the physical characteristics of model patients may cause a sense of shame, informing incidental findings, including their unique physical characteristics, in front of their peers could be against the expectations of examinees. Supplementary Data G provides further discussions on the view of female students on being model patients in Japan.

The study found that protecting students’ autonomy and ensuring confidentiality are priority tasks. However, further research is required to develop a procedure to obtain consent and a PPE policy that fits Japanese culture to address these issues. It should be noted that this study has several limitations. First, the unwillingness to undergo examination of certain body parts may differ according to the gender of the examiner [29]. However, we believe that filling an informed consent form regardless of the gender of the examiner is crucial for students due to practical limitations and educational values. Doing so would provide students with opportunities to empathize with patients undergoing examination by a medical doctor of the opposite gender. It would also reduce the administrative task of grouping. Second, we did not collect data on the details of abnormal findings. Therefore, not only medical problems but also unique physical characteristics may have been reported due to various interpretations of the abnormal findings.

The educational implication of this study is that medical students expect high levels of autonomy and confidentiality in attending PPEs as model patients. Thus, developing a PPE policy appropriate to Japanese culture and organizing induction sessions for new students with senior students demonstrating PPE and the educational benefit of being a model patient may contribute to the establishment of a PPE practice environment that maximizes satisfaction of and learning effect on students.

## Conclusions

We conclude that providing opportunities to all medical students to be model patients regardless of gender combined with the introduction of a procedure for obtaining consent and a PPE policy is a key to creating a student-friendly practice environment for PPEs. The findings suggest that the management of and measures for abnormal findings in the case of a breach in confidentiality also need to be included in the PPE policy.

### Electronic supplementary material

Below is the link to the electronic supplementary material.

Supplementary Material 1

## Data Availability

The datasets supporting the conclusions of this article are included within the article and its additional files.

## Abbreviations

PPEs: peer physical examinations
TMDU: Tokyo Medical and Dental University

## References

[1] Towle A (1991). Critical thinking: the future of Undergraduate Medical Education.

[2] Olson LG, Hill SR, Newby DA (2005). Barriers to student access to patients in a group of teaching hospitals. Med J Aust.

[3] Wearn A, Bhoopatkar H (2006). Evaluation of consent for peer physical examination: students reflect on their clinical skills learning experience. Med Educ.

[4] O’Neill PA, Larcombe C, Duffy K, Dorman TL (1998). Medical students’ willingness and reactions to learning basic skills through examining fellow students. Med Teach.

[5] Chang EH, Power DV (2000). Are medical students comfortable with practicing physical examinations on each other?. Acad Med.

[6] Rees CE, Wearn AM, Vnuk AK, Sato TJ (2009). Medical students’ attitudes towards peer physical examination: findings from an international cross-sectional and longitudinal study. Adv Health Sci Educ Theory Pract.

[7] Gupta V, Begum Y, Singh A, Agrawal D. Perception of medical students towards teaching basic clinical skills in otorhinolaryngology through peer physical examination (PPE). J Educ Health Promot. 2023;12. 10.4103/jehp.jehp_1165_21 10.4103/jehp.jehp_1165_21 PMC10317272 37404916

[8] Sato TJ, Patten D, McLachlan JC (2009). Cultural barriers to the spread of clinical skills teaching methods. Int J Clin Ski.

[9] Tagawa M, Ichinose M, Tanabe M (2004). Physical examination training and gender of Japanese medical students: analysis of a questionnaire about peer training and instructors. Med Educ (Japan).

[10] Braunack-Mayer AJ (2001). Should medical students act as surrogate patients for each other?. Med Educ.

[11] Pols J, Boendermaker PM, Muntinghe H (2003). Incidence of and sequels to medical problems discovered in medical students during study-related activities. Med Educ.

[12] Koehler N, Currey J, McMenamin C (2014). What should be included in a peer physical examination policy and procedure?. Med Sci Educ.

[13] Koehler N, McMenamin C (2014). The need for a peer physical examination policy within Australian medical schools. Med Teach.

[14] AMA Principles of Medical Ethics. Medical students practicing clinical skills on fellow students, Opinion 9.2.5. https://code-medical-ethics.ama-assn.org/ethics-opinions/medical-students-practicing-clinical-skills-fellow-students (2022). Accessed 2023-10-22.

[15] Das M, Townsend A, Hasan MY (1998). The views of senior students and young doctors of their training in a skills laboratory. Med Educ.

[16] Medical Education Model Core Curriculum Coordination Committee, Medical Education Model Core Curriculum Expert Research Committee. Model core curriculum for medical education in Japan. AY 2016 Revision. 2016. https://www.mext.go.jp/content/20230323-mxt_igaku-000028108_00005.pdf. Accessed 2023-10-22.

[17] Nara N, Beppu M, Tohda S, Suzuki T (2009). The introduction and effectiveness of simulation-based learning in medical education. Intern Med.

[18] Akaike M, Fukutomi M, Nagamune M, Fujimoto A, Tsuji A, Ishida K (2012). Simulation-based medical education in clinical skills laboratory. J Med Invest.

[19] Krishnan DG, Keloth AV, Ubedulla S (2017). Pros and cons of simulation in medical education: a review. Int J Med Health Res.

[20] O’Brien BC, Harris IB, Beckman TJ, Reed DA, Cook DA (2014). Standards for reporting qualitative research: a synthesis of recommendations. Acad Med.

[21] von Elm E, Altman DG, Egger M, Pocock SJ, Gøtzsche PC, Vandenbroucke JP (2008). The strengthening the reporting of observational studies in epidemiology (STROBE) statement: guidelines for reporting observational studies. J Clin Epidemiol.

[22] Glaser B, Strauss A (1967). The discovery of grounded theory: strategies for qualitative research.

[23] Green J, Thorogood N, Edition. F, editor.).

[24] Creswell JW, Clark VLP (2017). Designing and conducting mixed methods research.

[25] Miles MB, Huberman AM, Saldana J (2019). Qualitative data analysis.

[26] Barnette JJ, Kreiter CD, Schuldt SS (2000). Student attitudes toward same-gender versus mixed-gender partnering in practicing physical examination skills. Eval Health Prof.

[27] Rees CE, Bradley P, McLachlan JC (2004). Exploring medical students’ attitudes towards peer physical examination. Med Teach.

[28] Collett T, Kirvell D, Nakorn A, McLachlan JC (2009). The role of living models in anatomy teaching: experiences from a UK medical school medical teacher. Med Teach.

[29] Burggraf M, Kristin J, Wegner A, Beck S, Herbstreit S, Dudda M (2018). Willingness of medical students to be examined in a physical examination course. BMC Med Educ.

