# Supplementary material for: Mixed-methods study of medical students’ attitudes toward peer physical examinations in Japan
Source: BMC Med Educ. 2024 Jun 20;24:681. doi: 10.1186/s12909-024-05635-4 (PMC11188174; doi:10.1186/s12909-024-05635-4)
Supplement: Supplementary file 1 — Supplementary Material 1 [file 12909_2024_5635_MOESM1_ESM.docx]

**Supplementary data A. Educational settings**

Under the nationwide curriculum reform in 2005 for medical schools in Japan, an objective structured clinical examination (OSCE) has become a requirement in the transition from preclinical to clinical years (Hirotaka et al., 2004). The purpose of introducing OSCE was to ensure that students acquire sufficient physical examination skills by the initiation of clinical clerkship. In TMDU, the students take the OSCE at the end of the fourth year, which is prior to starting clinical clerkship in fifth year. Therefore, PPEs begin during preclinical clerkship in the fourth year as a preparation for OSCE. PPEs for head, neck, thorax, abdomen, back, arms, shoulders, and legs have been incorporated in pre-clinical and clinical clerkship. In contrast, the training for the physical examination on breast, genitals, and inguinal or rectal areas are exclusively dependent on simulation-based learning.

The Institute of Education has been responsible for the curriculum arrangement for pre-clinical and clinical clerkships in TMDU. The student–teacher ratio during PPEs is expected to be 10:1. The teachers and the Institute of Education share the contents of PPEs through a syllabus. The academic affairs section organizes PPE groups of approximately 10 students each. In grouping, the ratio of men to women is expected to be the same as that of men to women in the entire class. During the PPE class, all students are encouraged to experience the role of doctor. PPEs are held in a clinical skills laboratory or a classroom in the university.

Reference

Hirotaka Onishi, Ichiro Yoshida. Rapid change in Japanese medical education. Med Teach. 2004;26(5):403-8. doi:10.1080/01421590412331270492.

**Supplementary data B.**

**Supplementary Figure 1. Sample eligibility criteria for quantitative data analysis**

5^th^ year students at TMDU as of July 2022 (113 participants)

6^th^ year students at TMDU as of July 2022 (106 participants)

Junior residents at TMDU Hospital as of December 2022 (190 participants)

Participants excluded due to:

- No response

(82 participants for 5^th^ students,

74 participants for 6^th^ students,

137 participants for junior residents)

- No answer to the name of alma mater

(2 participants for junior residents)

- Graduated from international medical school (1 participant for junior resident)

5^th^ year students at TMDU as of July 2022 (31patients)

6th year students at TMDU as of July 2022 (32 participants)

Junior residents at TMDU Hospital as of December 2022 (50 participants)

**Supplementary Figure 2. Sample eligibility criteria for qualitative data analysis**

5^th^ year students at TMDU as of December 2022 (113 participants)

6^th^ year students at TMDU as of December 2022 (106 participants)

Junior residents at TMDU Hospital as of December 2022 (190 participants)

Participants excluded due to:

- No response

(103 participants for 5^th^ students,

102 participants for 6^th^ students,

188 participants for junior residents)

5^th^ year students at TMDU as of July 2022 (10patients)

6th year students at TMDU as of July 2022 (4 participants)

Junior residents at TMDU Hospital as of December 2022 (2 participants)

**Supplementary Table 1. Characteristics of the Participants (Interviews)**

|  | Male | Female |
| --- | --- | --- |
| Fifth-year students | 6 | 4 |
| Sixth-year students | 1 | 3 |
| First-year junior residents | 1 | 1 |
| Second-year junior residents | 0 | 0 |
| Total (n = 16) | 8 (50%) | 8 (50%) |

**Supplementary data C. Questionnaire items**

**C-1. Questions for medical students**

1. What year are you in medical school?

□5th year

□6th year

2. What is your gender?

□Male

□Female

□Do not answer

3. Please tell us about your experience on physical examination training at Tokyo Medical and Dental University. Have you ever practiced physical examination with other students as part of the curriculum?

□Yes

□No

4. Have you ever practiced physical examination with other students (hereafter, PPEs) as part of the university curriculum?

□Yes

□No

5. What was the style of the PPEs?

□There was no faculty member in a supervisory position, and the students alone performed physical examination on simulation models.

□There was no faculty member in a supervisory position, and multiple students performed physical examination on one another.

□There was at least one supervisor, and students performed physical examinations on simulation models.

□There was at least one supervisor, and multiple students performed physical examinations with one another.

□Other ( )

6. Have you ever played the role of a patient in a physical examination that involved undressing?

□Yes

□No

7. Did you feel uncomfortable with being a patient that involves undressing in a PPE class?

□Not at all

□Rarely

□Sometimes

□Often

□Always

8. In a PPE class, have you experienced the role of a patient that involves exposure of the following areas?

□Head and neck

□Palm

□Arm/shoulder

□Upper body

□Lower body

□Thorax

□Abdomen

□Back

□Groin

□Lower Extremity/Lower Leg

□Knee

□Gynecological and urological areas

9. Assume that you are asked to be a patient for an examination that involves the exposure of the following areas in a PPE class. Which areas do you feel uncomfortable with?

□Head and neck

□Palm

□Arm/shoulder

□Upper body

□Lower body

□Thorax

□Abdomen

□Back

□Groin

□Lower Extremity/Lower Leg

□Knee

□Gynecological and urological areas

10. We would like to ask those who played the patient role in PPEs that required undressing. When you played this role, was your privacy protected?

□Very much

□Fairly much

□No

□Not at all

□I don't know.

11. Have you ever played the patient role that had to undress for an examination against your will?

□Not at all.

□Rarely.

□Sometimes.

□Often.

□All the time.

12. Do you feel resistance in performing a physical examination on a student whose gender is different from yours?

□Not at all.

□No, I don’t think so.

□Yes and No.

□Fairly much.

□Very much.

13. A view exists that practicing auscultation and echocardiography skills is not necessary for students if they practice them using simulators. Do you agree or disagree with this view?

□Agree □Disagree □Other ( )

14. Have you ever had any unpleasant experiences during the PPE class? If so, please specify.

_____________

15. During the PPE class, were there any behaviors of the instructor or students toward other students that were unbecoming of a professional? If so, please specify.

_____________

16. What did you or your supervisor do when you accidentally found abnormal findings on the body of a student that played the patient role?

□I pointed it out publicly on the spot.

□I informed the student of it secretly.

□There was no such situation, or I did not notice it.

□I don't remember or I don't know.

□Other( )

17. Prior to the PPE class, were you given guidelines regarding playing the role of the patient or did you sign a document confirming that you had a right to refuse to play the role of the patient?

□There were guidelines or I signed a document confirming my will.

□There were no guidelines and I never signed a document confirming my willingness.

□I do not remember.

18. Is there anything you would like to see improved about the PPEs?

□Introduction of a procedure (oral) for obtaining consent to play the role of a patient in a partial or full body examination.

□Introduction of a procedure (written) for obtaining consent to play the role of a patient in a partial or full body examination.

□Guarantee of the freedom to refuse to play the patient role regardless of gender.

□Ensure confidentiality.

□Establishment of a consultation service.

□Rule-based measures against a breach in confidentiality

□Other ( )

19. Questions for men. What do you think about women playing the role of a patient in a PPE class

□I think it is a good idea

□I don't think it is a good idea

□I don't know.

20. If you answered “I don’t think it is a good idea” to the abovementioned question, please tell us why.

_____________

21. Questions for women. What do you think about men playing the role of a patient in a PPE class?

□I think it is a good idea.

□I don’t think it is a good idea.

□I don't know.

22. If you answered “I don’t think it is a good idea,” please tell us why.

**C-2. Questions for junior residents**

1. How long have you worked as a physician?

□Less than 1 year 　□1 to 2 years 　□More than 2 years

2. What is your gender?

□Male 　□Female 　□Do not answer

3. Have you ever practiced physical examination with other students (hereinafter, PPEs) as part of the university curriculum when you were a medical school student?

□Yes □No □I don't remember.

4. What university did you graduate from? ( )

5. What year did you graduate from medical school? ( )

6. Do you think the PPEs were necessary for you to work as a medical doctor?

□I thought it was necessary when I was a student and I still think it is necessary now.

□I did not think it was necessary when I was a student, but I think it is necessary now.

□I thought it was necessary when I was a student, but I don't think it is necessary now.

□I did not think it was necessary when I was a student, and I don’t think it is necessary now.

□Other ( )

7. What was the style of the PPEs?

□There was no faculty member in a supervisory position, and students alone performed physical examination on simulation models.

□There was no faculty member in a supervisory position, and multiple students performed physical examinations on one another.

□There was at least one supervisor, and students performed physical examination on simulation models.

□There was at least one supervisor, and multiple students performed physical examinations with one another.

□Other ( )

8. Have you ever played the role of a patient in a physical examination that involved undressing during your school days?

□Yes

□No

9. Did you feel resistance in being a patient that involves undressing in a PPE class?

□Not at all.

□Rarely.

□Sometimes.

□Often.

□Always.

10. In a PPE class, have you ever experienced the patient role that involved exposure of the following areas:

□Head and neck.

□Palm.

□Arm/shoulder.

□Upper body.

□Thorax.

□Abdomen.

□Back.

□Groin.

□Lower Extremity/Lower Leg.

□Knee.

□Gynecological and urological areas.

11. Assume that you are asked to be the patient for an examination, which involves exposure of the following areas in a PPE class. Which areas do you feel resistance in?

□Head and neck.

□Palm.

□Arm/shoulder.

□Upper body.

□Chest.

□Abdomen.

□Back.

□Groin.

□Lower extremity/lower leg.

□Knee.

□Gynecological and urological areas

12. We would like to ask those who have played the role of a patient that required undressing in a PPE class: When you played the patient role, was your privacy protected?

□Very much.

□Fairly much.

□No, I don't think so.

□Not at all.

□I don't know.

13. Have you ever played the role of a patient who had to undress for an examination against your will?

□Not at all.

□Rarely.

□Occasionally.

□Often.

□All the time.

14. Do you feel resistance in performing a physical examination on a student whose gender is different from yours?

□Not at all.

□No, I don’t think so.

□Yes and no.

□Fairly much.

□Very much.

15. A view exists that practicing auscultation and echocardiography skills is not necessary for students if they practice them using simulators. Do you agree or disagree with this view?

□Agree □Disagree □Other ( )

16. Have you ever had any unpleasant experiences during the PPE class? If so, please specify.

____________

17. During the PPE class, were there any behaviors of the instructor or students toward other students that were unprofessional? If so, please specify.

___________________

18. What did you or your supervisor do when you accidentally found abnormal findings on the body of a student playing the role of a patient?

□I pointed it out publicly on the spot.

□I informed the student of it secretly.

□There was no such situation, or I did not notice it.

□I don’t remember, or I don’t know.

□Other ( )

19. Prior to the PPE class, were you given guidelines regarding playing the patient role or did you sign a document confirming that you had the right to refuse to play the patient role?

□There were guidelines or I signed a document confirming my will.

□There were no guidelines and I never signed a document confirming my willingness.

□I do not remember.

20. Is there any aspect you would like to see improved about the practice of PPEs?

□Introduction of a procedure (oral) for obtaining consent to play the patient role in a partial- or full-body examination.

□Introduction of a procedure (written) for obtaining consent to play the patient role in a partial- or full-body examination.

□Guarantee of freedom to refuse to play the patient role regardless of gender.

□Ensure confidentiality.

□Establishment of a consultation service.

□Rule-based measures against a breach in confidentiality.

□Other ( )

21.Question for men; What do you think about women playing the role of a patient in a PPE class?

□I think it is a good idea.

□I don't think it is a good idea.

□I don't know.

22. If you answered “I don’t think it is a good idea,” please tell us why.

______________

23. Questions for women. What do you think about men playing the patient role in a PPE class?

□I think it is a good idea.

□I don’t think it is a good idea.

□I don't know.

24. If you answered “I don’t think it is a good idea,” please tell us why.

_______________

**Supplementary data D. Interview guide for medical students and junior residents about their PPE experience**

Thank you for taking time out of your busy schedule to participate in our survey, which examines issues regarding PPEs in clinical practice at the School of Medicine from the perspective of human rights protection for medical students. My name is Nobutoshi Nawa, an associate professor in the Department of Global Health Promotion, and Emily Suzuki, adjunct lecturer in the Department of Medical Education Research and Development. We are co-researchers of this study. In training for physical examination skills, students take turns in patient and physician roles to acquire opportunities for performing PPEs. In particular, in PPEs that involves undressing, ensuring that students regardless of gender are given the “freedom not to play the role of a patient in an examination that involves undressing in front of other students” is important. This freedom is clearly stated in overseas policies, although such policies do not exist in Japan. Additionally, the procedure for incidental findings during PPEs remains unclear. We believe that developing policies that reflect the cultural background of Japan is necessary for improving the situation regarding the freedom of decision-making and privacy protection among medical students in Japan. Therefore, we would like to interview you to hear your experience during PPEs. In the interviews, please be frank with us, as all the opinions are valuable. The information obtained from the interviews will be anonymized for analysis and reported in a manner that will not identify individuals. We hope to use this information to improve future education. This research will also be conducted with the approval of the Ethics Review Committee. We would like to have the interviews recorded. We will transcribe the interview data, anonymize it, and then destroy it appropriately.

Questions

1. When did you experience pre-clinical and clinical clerkship as a student (Year_____ to year_____)?

2. Did you perform PPE in a group of several students?

3. If yes, what was the ratio of the male to female students in the group?

4. If you experienced PPEs with a group of other students, how were the groups determined?

(PROBING: Was there any effort in determining the ratio of men to women in the group? If so, please tell us how it was done.)

5. If you were participating in a physical examination as a patient in which your instructor or another medical student is acting as a doctor and recognizes a finding on your body that was suspected to be abnormal, how would you want them to respond to it? This scenario includes cases in which the finding is known to the student playing the patient. However, we assume that this student did not report the known finding to the instructor or the medical students participating in the exercise in advance.

＜Please answer freely. If none of the following options is applicable to you, please indicate alternatives.>

a. For educational purposes, I would like my classmate or instructor to point incidental findings in the presence of other students.

b. I would like to point out the problem individually with respect to my privacy.

c. I don’t want my classmate or instructor to inform me of the incidental findings, but I would like them to contact the department that is responsible for the student's health care.

d. I think the university should ask students about their preferences regarding the method of notification in the case of the incidental findings prior to PPEs and take actions based on the student’s wish.

e. I do not want my classmate or instructor to inform anyone about it, including me.

6. In the pre-survey, a few students have been informed about the incidental findings in the presence of other students. What do you think about their experience?

7. When you participated in the PPE class, what did you care about?

<You can answer freely. The answer will be categorized into a–d. >

a. Safety.

b. Accuracy of the procedure.

c. Privacy protection for the student playing the patient role

d. Other

8. Do you think your privacy was fully protected during the PPE?

–No

–Yes

If “no,” then please allow me to record your (respondent's) gender.

Were you playing the role of a patient?

(If not, ask the gender of the student who played the patient role)

When you say that privacy was not fully protected, do you mean that privacy for the patient was fully protected?

If so, in what specific ways did you think privacy was not fully protected?

What do you think should have been done to protect the privacy of the patient?

9. Have you ever been asked to play the role of a patient against your will?

-Yes

Why? (PROBING: External pressure, No one else was available, etc.)

When you were forced to play the role of a patient against your will, what did you honestly want at that time?

-No

(PROBING: In the pre-survey, a few students were forced to play the patient role. What do you think about the potential reasons for such situations?

What do you think such students should have done?

10 A view exists that practising physical examination with one another is not necessary for students if they practise using simulators. Do you agree or disagree with this view? Please tell us why.

11. Do you think that the experience of playing the patient role is meaningful for a medical student?

These are all the questions we have for you. If you have any thoughts or questions regarding this matter, please do not hesitate to contact us. Thank you very much for your valuable time today.

**Supplementary data E.** **Supplementary Table. Reported unwillingness of participants**

|  | Male participants  (n=61)^a^ | Female participants  (n=46) | Total  (n=107) | df | p-value |
| --- | --- | --- | --- | --- | --- |
| Head and neck | 1 (1.6%) | 3 (6.5%) | 4 (3.7%) | 1 | 0.187 |
| Hand, arm and shoulder | 1 (1.6%) | 11(23.9%) | 12 (11.2%) | 1 | **<0.001** |
| Upper body | 10 (16.4%) | 36 (78.3%) | 46(43.0%) | 1 | **<0.001** |
| Thorax | 13 (21.3%) | 43 (93.5%) | 56 (52.3%) | 1 | **<0.001** |
| Abdomen | 14 (23.0%) | 34 (73.9%) | 48 (44.9%) | 1 | **<0.001** |
| Back | 5 (8.2%) | 35 (76.1%) | 40 (37.4%) | 1 | **<0.001** |
| Groin | 53 (86.9%) | 43 (93.5%) | 96 (89.7%) | 1 | 0.266 |
| Lower leg | 8 (13.1%) | 19 (41.3%) | 27 (25.2%) | 1 | **<0.001** |
| Knee | 1 (1.6%) | 7 (15.2%) | 8 (7.5%) | 1 | **0.008** |
| Gynecological and urological areas | 45 (73.8%) | 43 (93.5%) | 88 (82.2%) | 1 | **0.008** |

Bold values indicate *p* < 0.05

^a^Six participants did not respond to this question.

**Supplementary data F. Result for unwillingness by body region**

The Supplementary table demonstrated that less than 10% of the participants expressed unwillingness to undergo an examination of the head, neck, and knee. In contrast, a high prevalence of unwillingness was observed for the examination of gynecological and urological areas (82.2%) and groin (89.7%). Additionally, unwillingness among the women was significantly higher than that of the men in the examination of a hand, arm and shoulder, the upper body, a thorax, an abdomen, the back and lower legs (*p* < 0.001), knees, and gynecological and urological areas (*p* = 0.008).

**Supplementary data G. Further discussion of the view of the female students on being model patients**

Providing students with the equal chance of being model patients regardless of gender would secure a model subject with a reduced burden for men and an increased learning benefit for women. However, the majority of the female participants indicated reluctance to being model patients. As shown in the significantly higher levels of unwillingness to be examined as a patient for the hand, arm, shoulder, upper body, thorax, abdomen, back, and lower leg among the female participants in comparison with the men, exposing body parts in front of peers may lead to discomfort that outweighs the learning motivation of among female students in the context of the Japanese culture. This finding implies that a provision of attire for female students, as included in a PPE policy, may not be useful for increasing the level of privacy protection for female students in Japan, in contrast to the PPE guideline of the University of Queensland, Australia.

References

The University of Queensland, Australia. MD Program Phase 1: Peer Physical Examination Guidelines 2020.
